# Environmental variables drive the latitudinal decline in diversity and functional guild distribution of seed endophytic fungi in the invasive plant *Bidens frondosa*

**DOI:** 10.3389/fmicb.2026.1942937

**Published:** 2026-09-23

**Authors:** Lifeng Zhou, Li’ang Zheng, Meng Xiao, Cheng Chen, Hongyuan Zheng

**Affiliations:** 1 Yellow River Conservancy Technical University, Kaifeng, China; 2 State Key Laboratory of Crop Stress Adaptation and Improvement, School of Life Sciences, Henan University, Kaifeng, China; 3 State Key Laboratory of Cotton Bio-breeding and Integrated Utilization, School of Life Sciences, Henan University, Kaifeng, China

**Keywords:** annual weed, *Bidens frondosa*, climate, latitudinal gradient, seed endophytic fungi, soil

## Abstract

Seed endophytic fungi are increasingly recognized as key determinants of plant fitness, yet the ecological processes governing their biogeographic patterns remain poorly understood. Here, we investigated the diversity, composition, and putative functional guilds of seed-associated fungal communities in the invasive plant *Bidens frondosa* along a latitudinal gradient from 28 to 41°N in China. Alpha diversity declined significantly with latitude, while beta diversity showed strong latitudinal structuring, with latitude explaining 45.7% of community variation. Community composition shifted from Sordariomycetes dominance at low latitudes to Saccharomycetes dominance at high latitudes, accompanied by a functional transition from saprotroph dominated to saprotroph-symbiotroph dominated guilds. Differential abundance analysis revealed that plant-pathogenic taxa (e.g., *Fusarium*) were enriched at low latitudes, whereas the cold-adapted yeast *Mrakia* dominated high-latitude populations. Distance-based redundancy analysis identified soil pH, cation exchange capacity, and soil organic carbon as the primary environmental filters structuring both taxonomic and functional community variation. In contrast, all climatic variables were excluded from the final models. These findings demonstrate that soil chemistry, rather than climate factors, acts as the dominant selective force associated with seed endophytic fungal communities along latitudinal gradients. This work provides mechanistic insights into the biogeographic organization of plant-microbe symbioses and highlights the potential role of seed-associated fungi in facilitating plant invasion across climatic gradients.

## 1 Introduction

Biological invasions threaten biodiversity and ecosystem health worldwide, causing substantial environmental and economic losses (Diagne et al., 2021; Roy et al., 2024; Thakur et al., 2025). Rapid climate change further alters the dispersal and invasion ranges of alien species (Feng et al., 2022; Sun et al., 2022). Elucidating the mechanisms that enable invasive plants to successfully establish and expand across large latitudinal gradients is therefore critical for understanding the drivers of invasion success (Xiao et al., 2019; Chen et al., 2021).

Seed endophytic fungi are vertically transmitted across generations and influence germination, seedling establishment, and early plant health (Al-Ani, 2019a; Matsumoto et al., 2021; Hajji-Hedfi et al., 2024; Qi et al., 2026). Despite their functional significance, seed endophytic microbiome remains far less studied than those of the rhizosphere and phyllosphere (Shade et al., 2017). This knowledge gap is especially pronounced for invasive plants, where seed-associated microbes may play a pivotal role in facilitating adaptation to novel environments and range expansion (Jeong et al., 2021). Characterizing the composition, functions, and core microbiomes of seed endophytes in invasive plants is therefore essential for understanding species environment coevolution during invasion.

Latitudinal gradients provide a natural experimental platform for testing the effects of environmental filtering on microbial community assembly (Liu et al., 2020). Both climatic conditions (temperature, precipitation) and soil properties (pH, organic matter, nutrients) vary systematically with latitude and act as hierarchical filters shaping microbial communities (Zhou et al., 2021; Huang et al., 2024; Nguyen et al., 2024). Soil serves as an important source pool for endophytic fungi, and its physicochemical properties indirectly shape seed communities through a soil inoculation effect (Tabassum et al., 2024). Thus, climate and soil jointly determine the assembly of seed-associated microbiomes along latitudinal clines.

For invasive plants, seed traits including germination characteristics often exhibit adaptive variation along latitudinal gradients (Liu et al., 2016; Xiao et al., 2019, Zhou et al., 2021; Yan et al., 2025). However, whether this adaptive variation along latitudinal gradients extends to the composition and function of seed endophytic microbial communities remains unknown. Addressing this question is critical for understanding how microbes may assist host plants in adapting to different climatic conditions during the invasion process.

To test these predictions, we collected seeds of the annual plant *Bidens frondose* from four populations spanning 28–41°N in China and conducted ITS gene sequencing to characterize fungal seed endophytes. Native to temperate North America, *Bidens frondose* is a pioneer species that has invaded a broad range from subtropical to cold temperate zones across Eurasia, threatening agricultural production and biodiversity in its invasive ranges (Khapugin et al., 2022). Specifically, we tested whether: (1) seed endophytic fungal diversity and composition vary along a latitudinal gradient; (2) taxonomic composition and FUNGuild-predicted functional guilds shift systematically with latitude; and (3) soil properties versus climatic factors differentially explain the observed patterns.

## 2 Materials and methods

### 2.1 Study organisms and sample collection

In this study, mature seeds of *Bidens frondose* were collected from four geographically distinct populations along a latitudinal gradient in China (see Table 1). Sampling was conducted between late October and early December 2025, corresponding to the period of seed maturity for each population. Within each population, seeds were obtained from 10 to 20 individual plants, with a minimum spacing of 10 m between plants to ensure genetic and micro-environmental independence. From these, undamaged and fully mature seeds from approximately three plants were pooled to form one biological replicate. Subsequently extracted DNA was obtained independently from each pooled sample, yielding three biological replicates per population and a total of 12 samples. Following collection, seeds were air-dried under natural conditions and subsequently stored at 4°C until further analysis.

**TABLE 1:** Climatic and soil variables of *Bidens frondose* seed collection sites across different latitudes.

Population	Jilin	Hebei	Hubei	Hunan
Longitude (° E)	110.08	115.15	113.74	123.60
Latitude (° N)	28.66	30.30	36.17	41.95
MAT (° C)	14.99	17.06	10.72	7.61
TS (° C)	795.65	902.82	988.32	1294.08
MAP (mm)	1460.00	1366.00	621.00	661.00
PS (mm)	58.64	56.55	103.12	102.88
PWQ (mm)	571.00	547.00	376.00	415.00
CEC (cmol(+)/kg)	233.00	206.00	212.00	279.00
SN (g/kg)	578.00	213.00	446.00	228.00
SOC (%)	566.00	200.00	523.00	310.00
pH	55.00	62.00	68.00	64.00

Climatic factors: MAT, Mean Annual Temperature; TS, Temperature Seasonality; MAP, Mean Annual Precipitation; PS, Precipitation Seasonality; PWQ, Precipitation of Wettest Quarter. Soil properties: CEC, Cation Exchange Capacity; SN, Soil Nitrogen; SOC, Soil Organic Carbon; pH.

### 2.2 Environmental variates

To characterize the abiotic context of the study populations, key climatic and soil variables were examined for each *B. frondosa* seed-collection site along the latitudinal gradient. Climatic factors included mean annual temperature (MAT), temperature seasonality (TS), mean annual precipitation (MAP), precipitation seasonality (PS), and precipitation of the wettest quarter (PWQ). Soil properties comprised cation exchange capacity (CEC), total nitrogen (TN), soil organic carbon (SOC), and pH.

Climate data (MAP, MAT, etc.) for each site were obtained from WorldClim at a spatial resolution of 2.5 arc-minutes, representing long-term averages for the period 2000–2018. Corresponding soil parameters (CEC, TN, SOC, and pH) were extracted from SoilGrids^1^ at a depth of 0–5 cm, which reflects the surface conditions relevant to seed development and maturation. A summary of the site-specific environmental data is provided in Table 1.

### 2.3 Surface sterilization of seeds and DNA extraction

Seeds were subjected to surface sterilization prior to molecular analysis. For each sample, approximately 100 mg of seeds were placed in a sterile 10 mL centrifuge tube and sequentially treated as follows: immersion in 70% ethanol for 1 min, followed by agitation in 1.5% (v/v) sodium hypochlorite solution on a platform shaker for 10 min. Subsequently, seeds were rinsed three times with sterile double-distilled water (ddH_2_O) and air-dried under aseptic conditions on a clean bench. The protocol follows established seed endophyte methodologies, relying on standard surface sterilization procedures (Zhang et al., 2022; Chen et al., 2024).

After drying, the sterilized seeds were transferred to a 2.0 mL microcentrifuge tube, finely pulverized in liquid nitrogen using sterile pestles, and stored at −80 °C until DNA extraction. Total genomic DNA was extracted from approximately 50 mg of homogenized seed powder using the DNeasy PowerSoil Pro Kit (QIAGEN, Germany), strictly following the manufacturer’s instructions. DNA concentration and purity were assessed spectrophotometrically with a NanoDrop One instrument (Thermo Fisher Scientific, Waltham, MA, United States). Finally, DNA samples were eluted in ddH_2_O and stored at −20 °C for subsequent analysis.

### 2.4 Fungal ITS amplification and sequencing

A total of 12 seed-pooled samples were PCR-amplified and sequenced. The fungal internal transcribed spacer (ITS) region was amplified using the primer pair ITS1F (5′-CTTGGTCATTTAGAGGAAGTAA-3′) and ITS2 (5′-GCTGCGTTCTTCATCGATGC-3′). PCR amplification was performed using the KAPA HiFi HotStart ReadyMix (Roche, Basel, Switzerland) according to the manufacturer’s instructions. The thermal cycling conditions consisted of an initial denaturation at 95°C for 3 min, followed by 35 cycles of denaturation at 95°C for 30 s, annealing at 52°C for 30 s, and extension at 72°C for 30 s, with a final extension at 72°C for 5 min. PCR products were verified by electrophoresis on a 1.5% agarose gel and subsequently purified using AMPure XP beads (Beckman Coulter, Brea, CA, United States) to remove primer dimers and nonspecific fragments. Sequencing libraries were constructed using the NEBNext Ultra II DNA Library Prep Kit (New England Biolabs, Ipswich, MA, United States) following the manufacturer’s instructions, including end repair, A-tailing, adapter ligation, and indexing PCR. Library concentration was measured with a Qubit 4.0 Fluorometer (Invitrogen, Carlsbad, CA, United States), and fragment size distribution was assessed using an Agilent 2100 Bioanalyzer (Agilent Technologies, Santa Clara, CA, United States). Equimolar pooled libraries were subjected to paired-end sequencing (250 bp) on an Illumina MiSeq platform (Illumina, San Diego, CA, United States) at Meige Gene Technology Co., Ltd. (Guangdong, China). The resulting paired-end reads were designed to overlap for subsequent merging and downstream bioinformatic analysis.

### 2.5 Bioinformatic processing of fungal ITS data

Bioinformatic processing of the fungal ITS sequencing data was performed in QIIME2 (version 2022.2) (Bolyen et al., 2019). Paired-end reads were merged using the q2-vsearch plugin, and primer sequences were trimmed using the q2-cutadapt plugin. The DADA2 plugin (Callahan et al., 2016) was employed for quality filtering, denoising, error correction, chimera removal, and the generation of amplicon sequence variants (ASVs). Singletons (ASVs occurring in only one sample) were removed to obtain a final feature table for downstream analysis. Taxonomic assignment of fungal ASVs was performed using a pretrained naive Bayes classifier against the UNITE database (version 8.0) for fungal ITS sequences (Abarenkov et al., 2023). Relative abundances of fungal taxa at the class level were calculated for subsequent analyses.

### 2.6 Statistical analysis

#### 2.6.1 Basic diversity analysis

Microbial community complexity was evaluated by computing the observed species richness and Shannon diversity index for the fungal ITS dataset utilizing the VEGAN package. Before estimating diversity, sequence data were standardized to 76,125 reads per sample. Beta diversity, which reflects variations in community composition among samples, was analyzed through Principal Coordinates Analysis (PCoA) based on Bray-Curtis dissimilarity matrices. Statistical assessment of differences in fungal community structure along the latitudinal gradient was conducted using permutational multivariate analysis of variance (PERMANOVA) with 999 permutations, implemented with the “adonis2” function in the vegan package.

#### 2.6.2 Functional guild prediction and latitudinal pattern analysis

Putative fungal ecological guilds were predicted from the ASV-derived taxonomic assignments using the FUNGuild database (Nguyen et al., 2016). Guild assignments with a confidence ranking of “Highly Probable” or “Probable” were retained for subsequent analyses, while those categorized as “Possible” were excluded to ensure robustness (Nguyen et al., 2016). The composition of fungal putative functional guilds across the latitudinal gradient was visualized using a stacked bar chart, which depicted the relative abundance of each guild per sample. To quantify the relationship between guild abundance and latitude, the relative abundance of each fungal guild was regressed against the latitude of the seed collection site using linear regression.

#### 2.6.3 Differential abundance analysis

To identify fungal taxa whose abundance varied significantly across latitudinal groups, differential abundance analysis was performed. The non-parametric Kruskal-Wallis rank-sum test was first applied to each taxon, followed by Benjamini-Hochberg false discovery rate (FDR) correction for multiple comparisons. A taxon was considered differentially abundant if it met two criteria: (1) an adjusted *p*-value (*q*-value) < 0.05, and (2) a minimum absolute log_2_ fold-change (LFC) > 1 when comparing its median relative abundance in the most abundant group to the combined median of all other groups. In cases where a taxon was absent in all comparator groups, an LFC of 10 was assigned to denote strong enrichment. To evaluate the robustness of these findings, a non-parametric bootstrap resampling procedure (1,000 iterations) was conducted, wherein samples were resampled with replacement within each group prior to re-applying the Kruskal-Wallis test, and the median bootstrap *p*-value along with its 95% confidence interval was calculated. Taxa with a median bootstrap *p* < 0.05 were retained for further analysis. All statistical analyses and visualizations were performed in R (v4.3.0) using the *vegan*, *dplyr*, *ggplot2*, and *pheatmap* packages.

#### 2.6.4 Correlations and distance-based redundancy analysis

Fungal community composition was characterized based on the relative abundance of fungal classes, the distribution of amplicon sequence variants (ASVs), and alpha diversity indices (Observed species and Shannon index). Relationships between these community metrics and the climate and soil variables were assessed using Pearson correlation analysis.

To evaluate the influence of environmental factors on the composition of seed endophytic fungal communities, a distance-based redundancy analysis (db-RDA) was performed using Bray-Curtis dissimilarity as the response variable (Pan et al., 2023). Prior to the analysis, a stepwise selection procedure based on the Akaike Information Criterion (AIC) was applied to the set of climatic and soil predictors to avoid multicollinearity and to build a parsimonious model. The significance of the overall model and of each canonical axis was tested using permutation tests (999 permutations). The individual and shared contributions of the selected soil properties to the explained variance were quantified using variance partitioning analysis. All statistical computations and visualizations were conducted in R (v4.3.0) with the primary support of the *vegan* and *ggplot2* packages.

## 3 Results

### 3.1 Diversity of seed fungal communities

Following quality filtering, denoising, and chimera removal, a total of 979,316 high-quality reads were generated from the 12 seed samples. These reads were subsequently clustered into 661 amplicon sequence variants (ASVs). To enable robust cross-sample comparisons, sequence libraries were rarefied to an even depth of 76,125 reads per sample, thereby minimizing biases arising from unequal sequencing effort and ensuring that subsequent diversity metrics reflect genuine biological variation.

Fungal alpha diversity within seeds was assessed using the Shannon diversity index and observed ASV richness. Both metrics exhibited significant linear declines with increasing latitude (Figures 1A,B). The Shannon index decreased from approximately 3.1 at lower latitudes (∼28°N) to below 1.5 at higher latitudes (∼41°N). Similarly, observed ASV richness declined from over 200 to fewer than 120 across the same gradient. These results indicate a pronounced latitudinal gradient in taxonomic diversity, with seed-associated fungal communities becoming progressively less rich and even toward higher latitudes.

**FIGURE 1:**
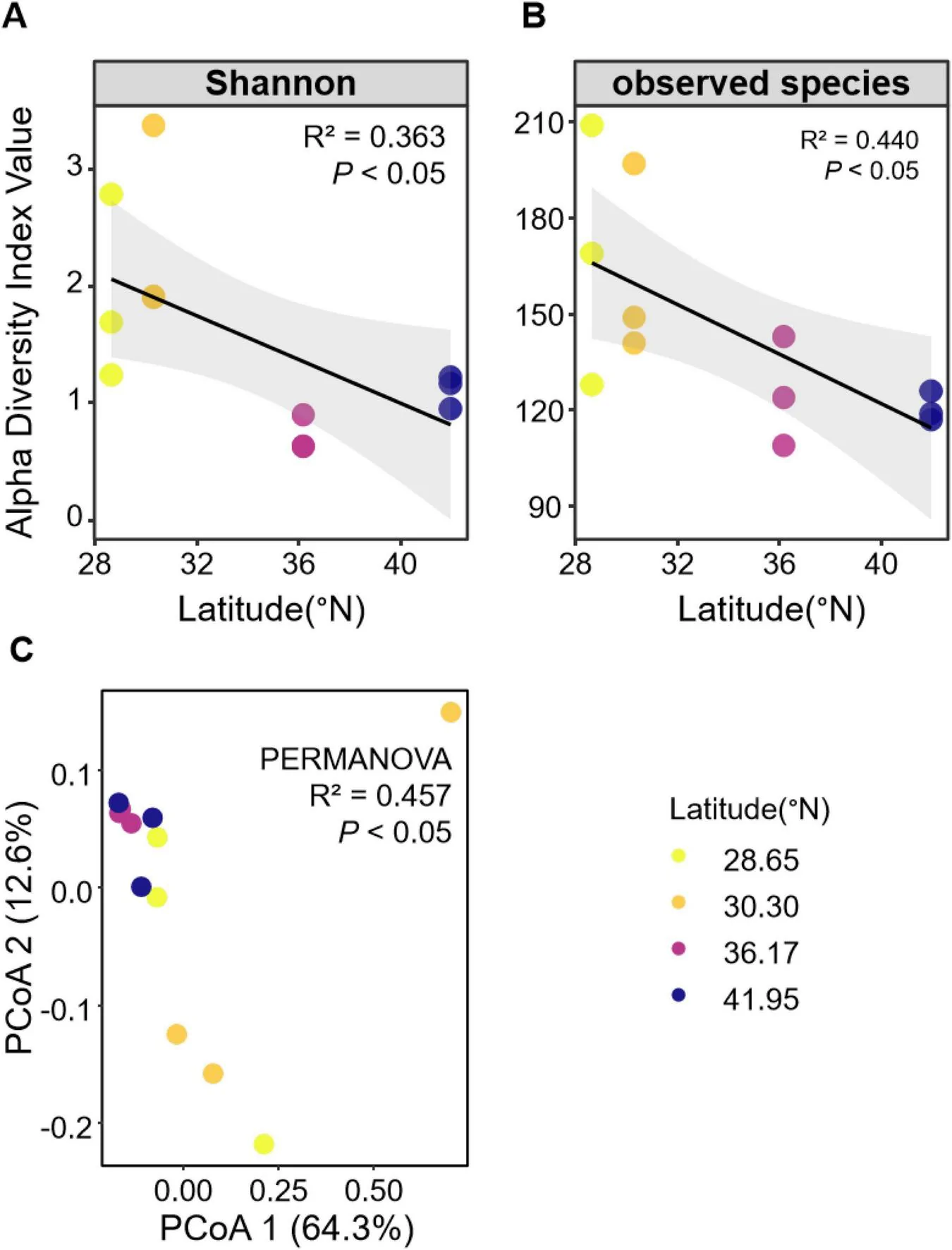
Latitudinal gradient patterns of seed endophytic fungal alpha and beta diversity. **(A)** Shannon diversity index and **(B)** observed species richness of seed endophytic fungi plotted against latitude (°N). **(C)** Principal Coordinates Analysis (PCoA) based on Bray-Curtis distances. Different colors represent sampling sites at different latitudes (28.65°N, 30.30°N, 36.17°N, and 41.95°N).

Beta diversity, representing compositional dissimilarity among communities, was visualized using principal coordinates analysis (PCoA) based on Bray-Curtis distances (Figure 1C). The first PCoA axis, explaining 64.3% of the total variance. Permutational multivariate analysis of variance (PERMANOVA) confirmed that latitude significantly structured community composition (*R*^2^ = 0.457, *P* < 0.05), indicating that geographic position explains approximately 45.7% of the variation in seed endophytic fungal assemblages.

### 3.2 Taxonomic and functional composition of seed endophytic fungal communities

Taxonomic classification revealed that the seed endophytic fungal communities were overwhelmingly dominated by the phylum Ascomycota, which accounted for a mean relative abundance of 98.36% across all samples. At the class level, fungal communities were dominated by Dothideomycetes and Sordariomycetes (Figure 2A). The relative abundance of Sordariomycetes decreased with increasing latitude (*R*^2^ = 0.385, *P* < 0.05; Figure 2B), whereas that of Saccharomycetes increased (*R*^2^ = 0.385, *P* < 0.05; Figure 2C), forming a clear latitudinal gradient in community composition. All other fungal classes were present at comparatively low abundances throughout the gradient and showed no consistent directional shifts with latitude (Supplementary Figure S2).

**FIGURE 2:**
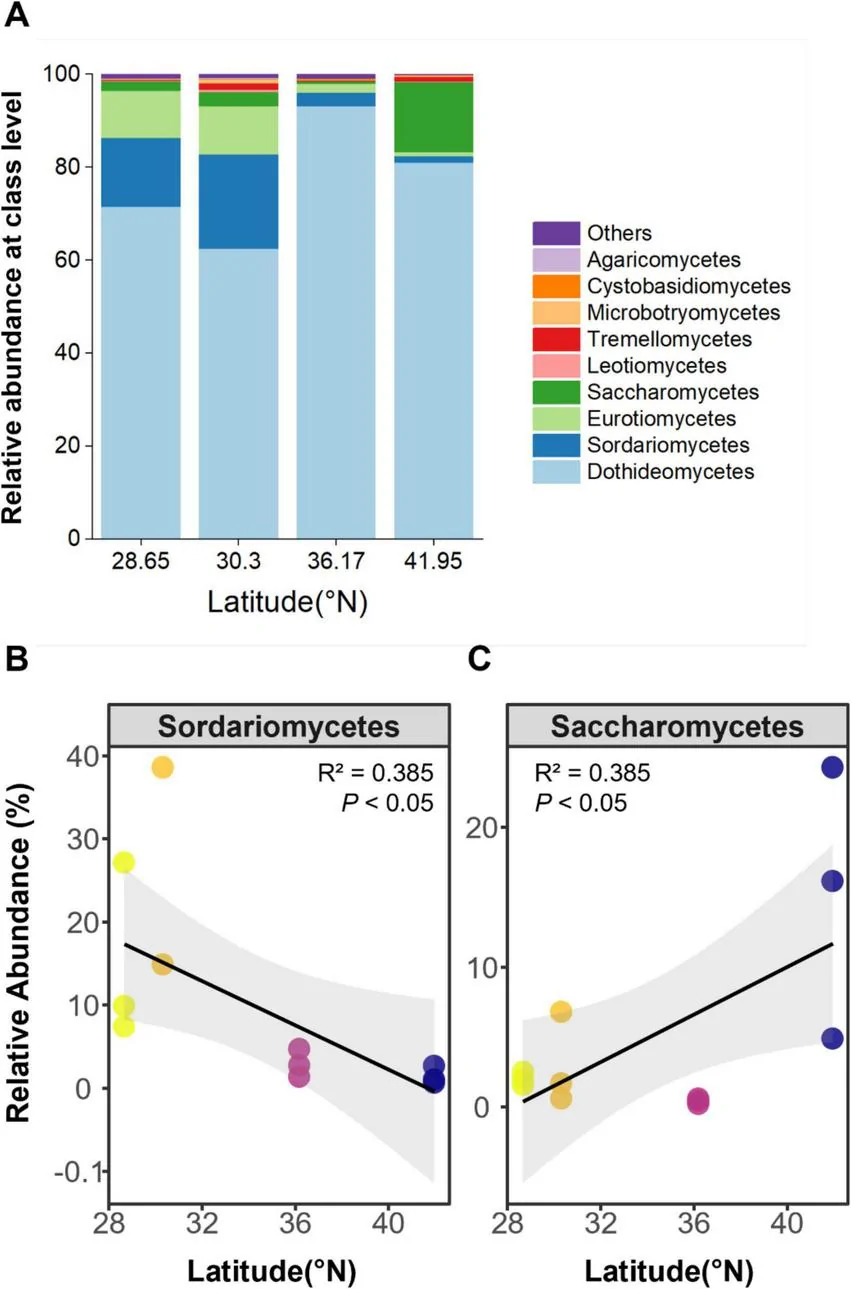
Class-level taxonomic composition and responses to latitude gradient. **(A)** The relative abundance of fungal classes across the latitudinal gradient. Latitudinal distribution patterns of the relative abundance of Sordariomycetes **(B)** and Saccharomycetes **(C)**.

Furthermore, functional guild analysis revealed a pronounced latitudinal reorganization in the putative ecological functions of seed endophytic fungi. Across all latitudes, the communities were dominated by the pathotroph-saprotroph guild, though its relative abundance declined significantly with increasing latitude (Figure 3A). Concurrently, the relative abundance of the saprotroph guild decreased markedly along the gradient (*R*^2^ = 0.334, *P* < 0.05; Figure 3B). A positive relationship with latitude was observed for the saprotroph-symbiotroph guild (*R*^2^ = 0.333, *P* < 0.05; Figure 3C). In contrast, other minor guilds, including symbiotrophs and pathotrophs exhibited no clear latitudinal pattern (Supplementary Figure S3).

**FIGURE 3:**
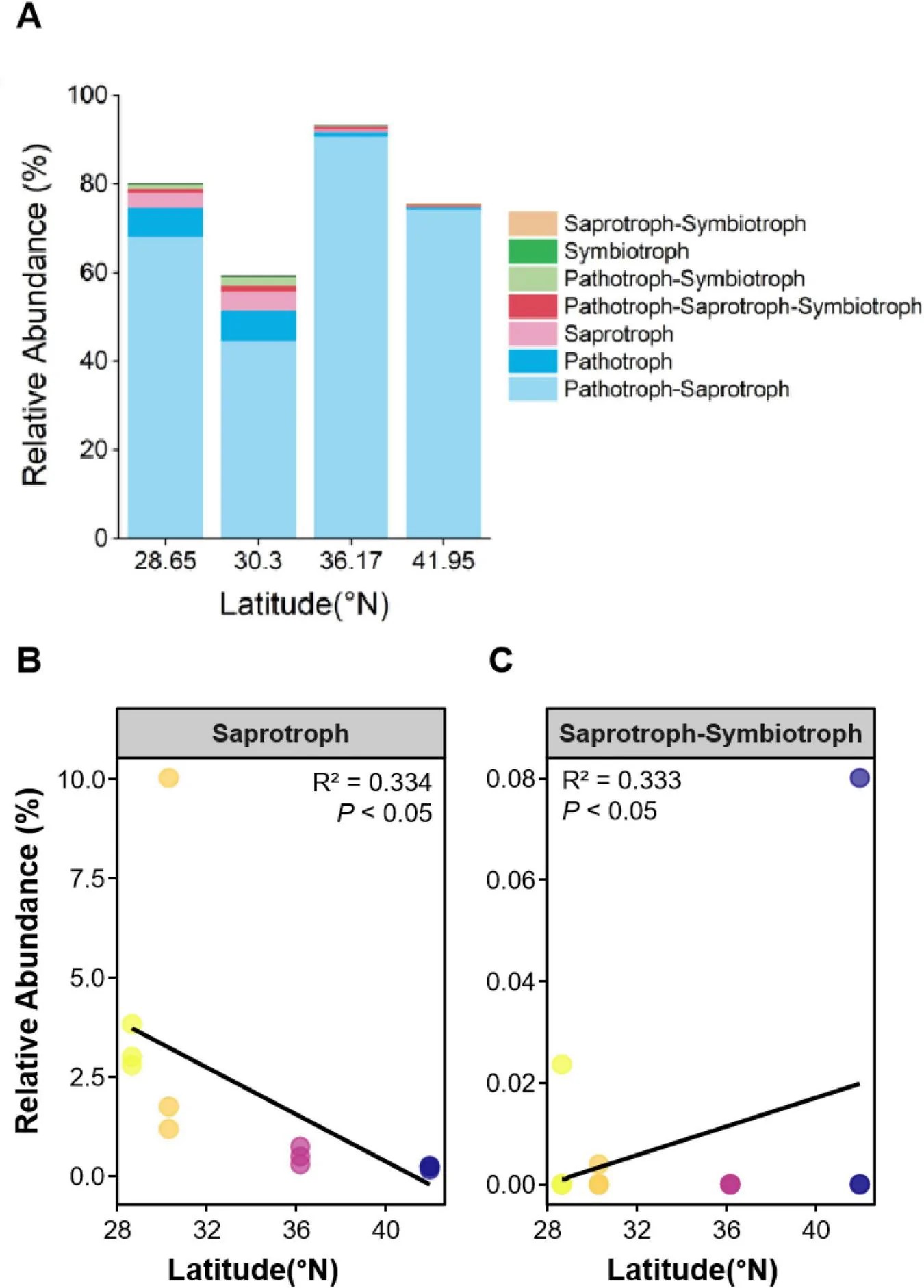
Trophic mode guild composition and responses to latitude gradient. **(A)** The relative abundance of fungal trophic modes across different latitudes. Latitudinal variation in the relative abundance of the **(B)** Saprotroph and **(C)** Saprotroph-Symbiotroph guild.

### 3.3 Differential abundance analysis

Differential abundance analysis identified 13 fungal ASVs whose relative abundance varied significantly across the four latitudinal groups (Figure 4 and Supplementary Table S3). The majority of these differential ASVs, which were taxonomically assigned to plant-pathogenic genera such as *Fusarium*, *Cercospora*, and *Stachybotrys*, were significantly enriched at the lowest-latitude site (28.65°N). Among these, ASV_7 (g_*Fusarium*) exhibited a particularly strong negative correlation with latitude, showing a sharp decline in relative abundance towards higher latitudes. In contrast, a distinct compositional shift characterized the highest-latitude site (41.95°N), which was uniquely defined by the significant enrichment of ASVs belonging to the genus *Mrakia*. Specifically, ASV_3 and ASV_69 (both classified as *Mrakia* spp.) displayed a pronounced positive relationship with latitude, becoming dominant members of the fungal assemblage in northern populations.

**FIGURE 4:**
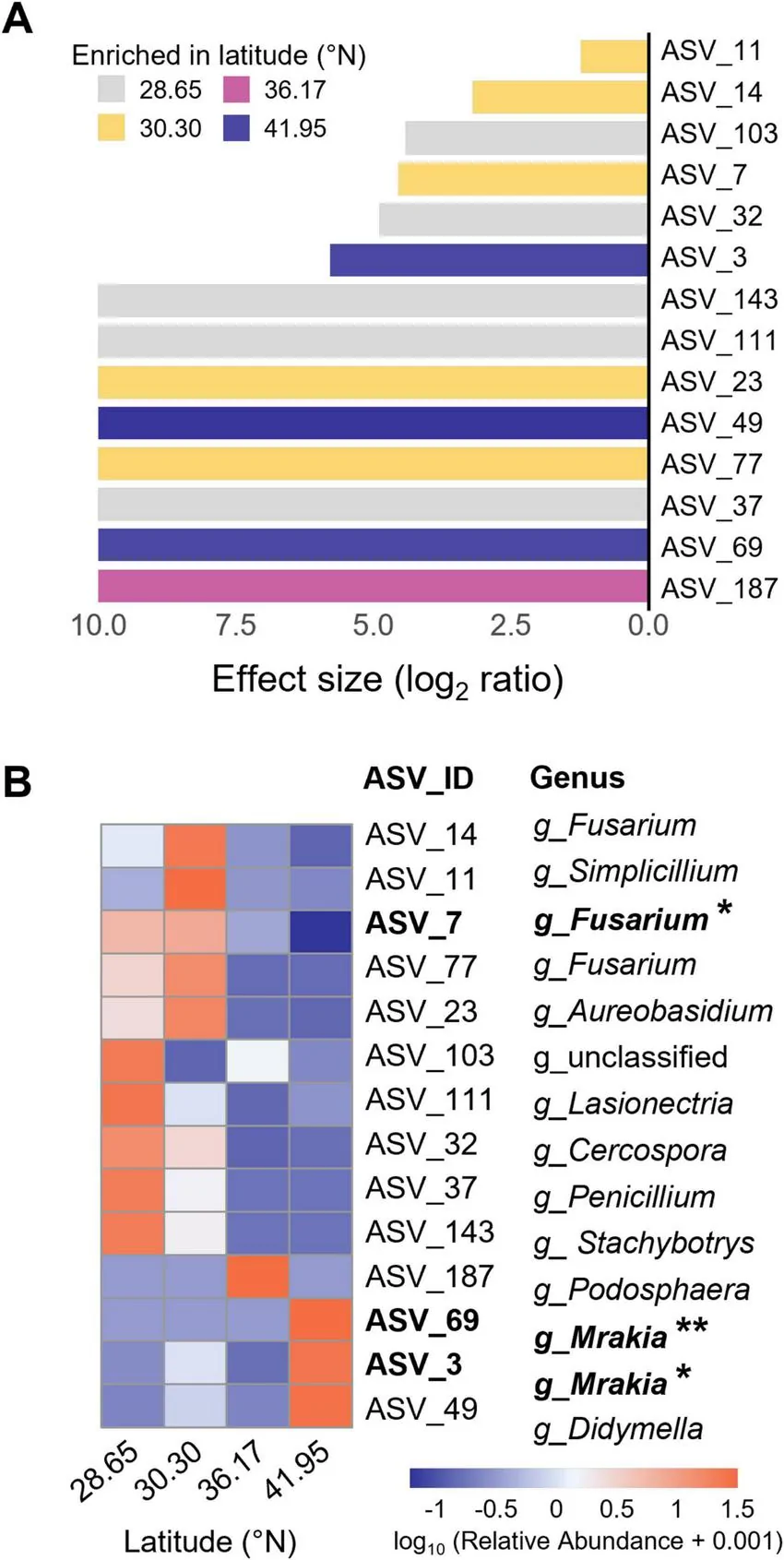
Identification and abundance of differentially ASVs along the latitudinal gradient. **(A)** the results of linear discriminant analysis effect size analysis (LEfSe) to identify biomarker ASVs associated with specific latitudes. Colors represent the latitude where the ASV was most abundant. **(B)** Heatmap illustrating the relative abundance of the differentially ASVs across the four sampling sites. Asterisks denote significant correlation with latitude for this ASV (*P < 0.05, **P < 0.01).

### 3.4 Environmental associations with fungal community structure

To elucidate the correlates of fungal community variation, we assessed the correlations between community metrics (alpha diversity, class-level abundances, guild-level abundances, and differential ASVs) and environmental variables (climate and soil properties) using Spearman’s correlation (Figure 1 and Supplementary Figures S4–S6). Alpha diversity indices (Shannon, Observed species) exhibited weak, scattered correlations with environmental factors (Figure 5A). In contrast, strong taxonomic and functional filtering was observed. The class Sordariomycetes was associated with higher temperatures and lower soil pH, whereas Saccharomycetes showed the opposite preference (Figure 5A). Functionally, pathogen-dominated guilds correlated with warmer, wetter conditions, while saprotrophs, including the cold-adapted yeast *Mrakia*, were linked to cooler climates (Supplementary Figure S4).

**FIGURE 5:**
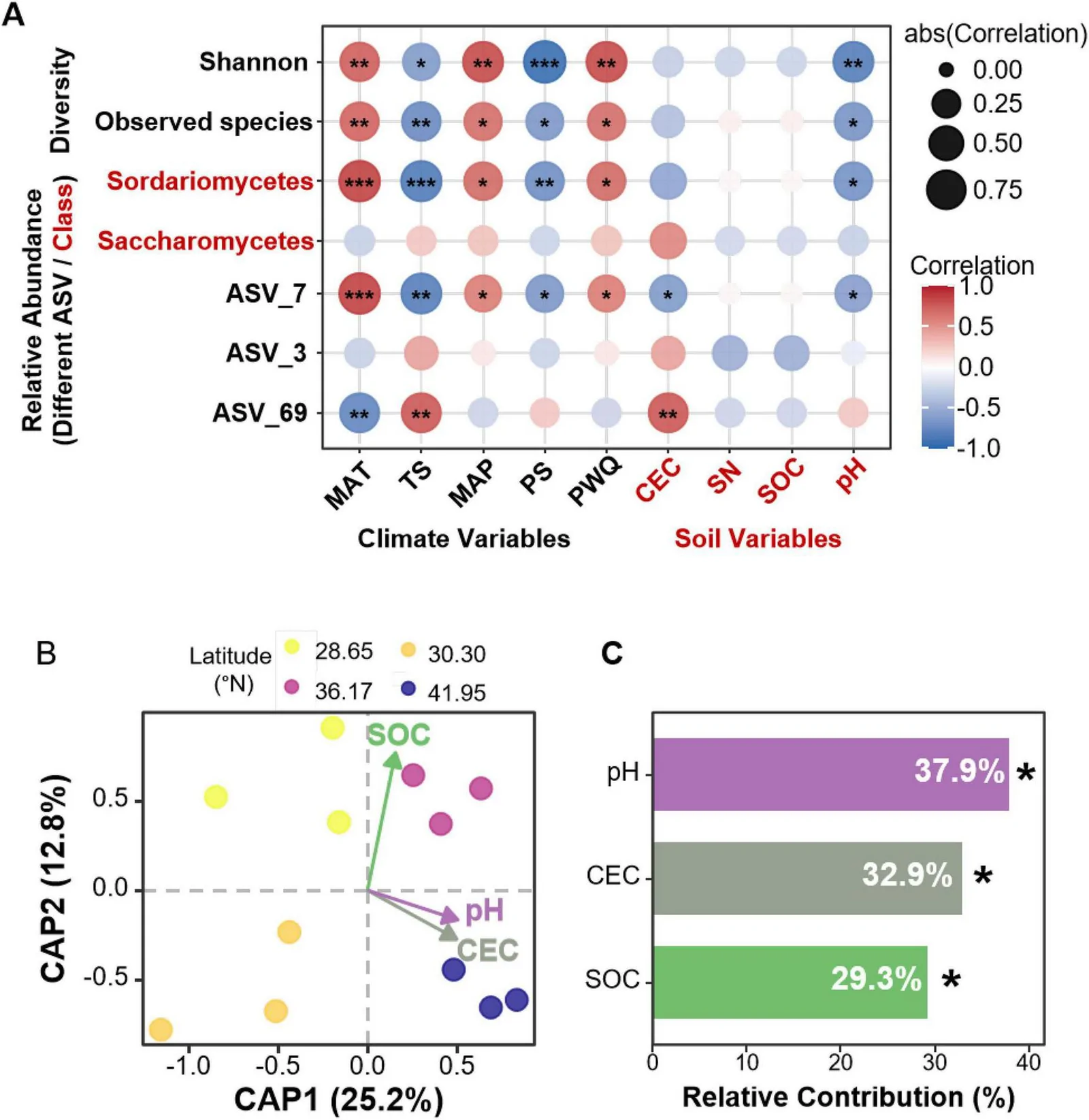
Effects of climatic and edaphic factors on seed fungal community structure. **(A)** Spearman’s correlation matrix between fungal community characteristics (alpha diversity indices, dominant classes, and differentially ASVs) and environmental variables (climate and soil properties). The size of the circles indicates the absolute correlation coefficient, and the color represents the correlation direction (red for positive, blue for negative). Climatic factors: MAT, Mean Annual Temperature; TS, Temperature Seasonality; MAP, Mean Annual Precipitation; PS, Precipitation Seasonality; PWQ, Precipitation of Wettest Quarter. Soil properties: CEC, Cation Exchange Capacity; SN, Soil Nitrogen; SOC, Soil Organic Carbon; pH. Asterisks indicate statistical significance: *P < 0.05, **P < 0.01, ***P < 0.001. **(B)** Distance-based redundancy analysis (db-RDA) triplot showing relationships among fungal communities (dots) and environmental variables (arrows). The ordination was constrained by soil properties (*R*^2^ = 0.246). **(C)** Bar plot indicating the relative contribution of environmental variables (pH, CEC, SOC) to the explained variance in the fungal community composition. Asterisks denote statistical significance (*P < 0.05) based on permutation tests.

To assess the relative influence of environmental factors on the seed endophytic fungal community, distance-based redundancy analysis (db-RDA) was applied to both taxonomic (ASV-based) and functional (FUNGuild-predicted) datasets (Figure 5A and Supplementary Figure S6). For taxonomic composition, the final model retained three soil variables: pH, cation exchange capacity (CEC), and soil organic carbon (SOC) (Figure 5B). It explained 24.6% of the total constrained variation, with the first two axes capturing 84.2% of the constrained inertia. Among the predictors, soil pH contributed most strongly (37.9%), followed by CEC (32.9%) and SOC (29.3%) (Figure 5C). Analysis of functional composition yielded a congruent pattern. The db-RDA model explained 41.5% of the constrained variation, with soil pH again emerging as the strongest correlate (52.6%), followed by SOC (32.5%) and CEC (14.9%) (Supplementary Figure S6). All climatic variables were excluded from the model during stepwise selection.

## 4 Discussion

In this study, we investigated the seed endophytic fungal communities of the invasive plant *Bidens frondosa* along a latitudinal transect in eastern China and identified their environmental correlates. Our results revealed a pronounced latitudinal gradient in fungal diversity. Dominant fungal classes showed contrasting latitudinal trends, with Saccharomycetes increasing and Sordariomycetes decreasing, accompanied by a functional shift from saprotrophs toward saprotroph-symbiotroph taxa at higher latitudes. Multivariate analysis identified soil chemical properties, particularly pH, as the strongest environmental correlates of community patterns. These findings extend macroecological biogeographic principles to the cryptic endophytic seed habitat and provide critical empirical evidence for understanding how invasive plants and their microbial symbionts co-adapt to large-scale environmental heterogeneity.

We demonstrate a significant latitudinal diversity gradient in seed endophytic fungal communities, with both alpha and beta diversity declining systematically toward higher latitudes. This pattern aligns with the classic latitudinal diversity gradient observed in many macro-organisms and some microbial groups (Xiao et al., 2021; Li et al., 2023; Huang et al., 2024), suggesting that the seed endophytic fungal assembly, despite occupying a specialized niche, follows broad macro-ecological rules. Furthermore, the significant compositional shift along latitude further indicates that environmental filtering not only reduces species richness but restructures the entire community (Xiao et al., 2021). Harsher abiotic conditions at higher latitudes, such as lower temperatures and shorter growing seasons, impose stronger environmental filtering and limit species survival. In contrast, greater precipitation variability and higher bioturbation intensity in low-latitude regions may facilitate the horizontal transfer of environmental fungi into seeds, enriching community composition. Together, these findings provide a novel case for understanding the macro-biogeographic rules that govern microbial communities within plant propagules.

Along the latitudinal gradient, the seed endophytic fungal community exhibited a consistent and coordinated shift in both taxonomy and putative function. Taxonomically, dominant taxa transitioned from Sordariomycetes at low latitudes to Saccharomycetes and the psychrotolerant yeast *Mrakia* at high latitudes. Within Sordariomycetes, the genus *Fusarium* which includes potentially plant-pathogenic species (Al-Ani, 2019b), was significantly enriched at low latitudes. Functionally, the community shifted from dominance by the saprotroph guild to increasing prominence of the saprotroph-symbiotroph guild, reflecting adaptive differentiation of ecological strategies along the environmental gradient (Zhang et al., 2021; Mertin et al., 2022). The enrichment of *Fusarium* at low latitudes may be favored by longer growing seasons, higher temperature and humidity, and stronger biotic interactions (Scherm et al., 2013; Maharachchikumbura et al., 2016; Al-Ani, 2019b). In contrast, the enrichment of *Mrakia* and Saccharomycetes at high latitudes is consistent with their known cold-adapted physiology (Dost et al., 2024; Li et al., 2025). The observed covariation between taxonomy and function demonstrates that the latitudinal gradient acts as an environmental filter, jointly associated with species composition and functional traits, mirroring directional selection of microbial life-history strategies by climatic severity.

Beyond climate, soil chemical properties, particularly pH, emerged as the strongest environmental correlates of seed endophytic fungal variation, outperforming climatic variables. Both db-RDA and correlation analyses consistently revealed soil pH, cation exchange capacity (CEC), and soil organic carbon (SOC) as the main predictors of community taxonomic and functional structure. This can be attributed to three mechanisms. First, soil serves as a fungal source pool, its microbial communities follow latitudinal gradients and supply seeds with inocula (Barbi et al., 2025; Rillig, 2026). Second, soil pH imposes physiological stress via nutrient availability, membrane permeability, and enzyme activity (Barrow and Hartemink, 2023). Third, soil chemistry modulates microbial interaction networks, indirectly filtering locally compatible taxa (Philippot et al., 2024). Although climatic factors correlated with seed endophytic diversity (Figure 5A and Supplementary Figures S3–S5), their exclusion from final models suggests that their effects are largely mediated through soil properties (Figure 5B and Supplementary Figure S6). Thus, seed community structure more directly reflects maternal rhizosphere conditions, from which seeds recruit microbes during development (Abdelfattah et al., 2023). Soil factors bridge large-scale climatic to seed microbial communities. Given the inherent collinearity among latitude, climate, and soil properties, we caution against inferring strict causality from the variance partitioning results.

This study is an exploratory biogeographic survey with four populations sampled in a single season. While the observed latitudinal patterns are statistically robust within this dataset, the limited number of sites and the absence of temporal replication prevent us from distinguishing true latitudinal signals from site-specific or interannual effects. Currently, the functional predictions rely on bioinformatic inference (FUNGuild), and the cold-adapted role of *Mrakia* is inferred from its known psychrotolerant physiology (Dost et al., 2024), but direct inoculation assays have not yet been conducted.

## 5 Conclusion

In summary, our results demonstrate that the seed endophytic fungal communities of *Bidens frondosa* exhibit a significant latitudinal trend, with soil chemical properties (pH, CEC, SOC) more strongly associated with community variation than climate alone. This highlights the need to prioritize soil chemistry in large-scale studies of the seed endophytic niche. These insights have implications for understanding the potential role of the seed microbiome in plant invasion across large spatial scales.

## Data Availability

The datasets presented in this study can be found in online repositories. The names of the repository/repositories and accession number(s) can be found at: https://www.ncbi.nlm.nih.gov/, PRJNA1479206.

## Footnotes

1
https://soilgrids.org
