# Supplementary material for: Environmental variables drive the latitudinal decline in diversity and functional guild distribution of seed endophytic fungi in the invasive plant Bidens frondosa
Source: Front Microbiol. 2026 Sep 23;17:1942937. doi: 10.3389/fmicb.2026.1942937 (PMC13642844; doi:10.3389/fmicb.2026.1942937)
Supplement: Supplementary file 1 [file Supplementary_file_1.docx]

Supplementary Table 1. Results of linear regression analysis showing the relationship between latitude and fungal community metrics (Diversity, Class-level and differential ASVs relative abundance, and Trophic mode guilds).

| Variables | | R^2^ | *P* |
| --- | --- | --- | --- |
| Diversity | Shannon | 0.363 | **0.038** |
|  | observed_species | 0.440 | **0.019** |
| Relative abundance at class level | Sordariomycetes | 0.385 | **0.031** |
|  | Saccharomycetes | 0.385 | **0.031** |
|  | Dothideomycetes | 0.125 | 0.260 |
|  | Eurotiomycetes | 0.317 | 0.057 |
|  | Tremellomycetes | 0.008 | 0.783 |
|  | Microbotryomycetes | 0.197 | 0.148 |
|  | Leotiomycetes | 0.078 | 0.378 |
|  | Cystobasidiomycetes | 0.014 | 0.711 |
|  | Agaricomycetes | 0.080 | 0.372 |
|  | Others | 0.174 | 0.178 |
| Trophic mode guild | Saprotroph | 0.334 | **0.049** |
|  | Saprotroph-Symbiotroph | 0.333 | **0.050** |
|  | Pathotroph-Saprotroph | 0.223 | 0.121 |
|  | Pathotroph | 0.315 | 0.058 |
|  | Pathotroph-Saprotroph-Symbiotroph | 0.221 | 0.123 |
|  | Pathotroph-Symbiotroph | 0.316 | 0.057 |
|  | Symbiotroph | 0.228 | 0.117 |
|  | Unassigned | 0.049 | 0.491 |
| Relative abundance of differential ASVs | ASV_23 | 0.256 | 0.093 |
|  | ASV_32 | 0.323 | 0.054 |
|  | ASV_49 | 0.218 | 0.126 |
|  | ASV_77 | 0.302 | 0.064 |
|  | ASV_7 | 0.373 | **0.035** |
|  | ASV_14 | 0.148 | 0.217 |
|  | ASV_111 | 0.267 | 0.086 |
|  | ASV_3 | 0.500 | **0.010** |
|  | ASV_37 | 0.248 | 0.099 |
|  | ASV_187 | 0.029 | 0.594 |
|  | ASV_11 | 0.055 | 0.461 |
|  | ASV_143 | 0.249 | 0.099 |
|  | ASV_69 | 0.509 | **0.009** |
|  | ASV_103 | 0.291 | 0.070 |

**Figure S1.** The relative abundance of fungal classes *Agaricomycetes, Cystobasidiomycetes, Dothideomycetes, Eurotiomycetes, Leotiomycetes, Microbotryomycetes*, Others, and *Tremellomycetes* across the four latitudes.

**Figure S2.** The relative abundance of fungal ecological putative functional guilds across the four latitudes.

**Figure S3. Correlation between fungal class-level relative abundances and environmental variables.** Climatic factors: MAT, Mean Annual Temperature; TS, Temperature Seasonality; MAP, Mean Annual Precipitation; PS, Precipitation Seasonality; PWQ, Precipitation of Wettest Quarter. Soil properties: CEC, Cation Exchange Capacity; SN, Soil Nitrogen; SOC, Soil Organic Carbon; pH. The size of the circles indicates the absolute value of the correlation coefficient, and the color represents the correlation direction (red for positive, blue for negative). Asterisks indicate statistical significance: **P*< 0.05, ***P*< 0.01, ****P*< 0.001.

**Figure S4.** Correlation between fungal guild-level relative abundances and environmental variables.

**Figure S5.** Correlation between the relative abundances of differentially ASVs and environmental variables.

**Figure S6.** Constrained Correspondence Analysis (CCA) and environmental factor contribution. **(A)** CCA triplot showing the relationships among seed fungal community samples, environmental variables (arrows), and colored dots representing different latitudes. **(B)** Bar plot indicating the relative contribution of environmental variables (pH, SOC, CEC) to the explained variance in the fungal community composition. Asterisks denote statistical significance (**P*< 0.05) based on permutation tests.

**Figure S1.**

**Figure S2.**

**Figure S3.**

**Figure S4.**

**Figure S5.**

**Figure S6.**
